# Modulation of Inflammatory Responses by Wnt/β-Catenin Signaling in Dendritic Cells: A Novel Immunotherapy Target for Autoimmunity and Cancer

**DOI:** 10.3389/fimmu.2016.00460

**Published:** 2016-10-27

**Authors:** Amol Suryawanshi, Raghu K. Tadagavadi, Daniel Swafford, Santhakumar Manicassamy

**Affiliations:** ^1^Cancer Immunology, Inflammation, and Tolerance Program, Georgia Cancer Center, Augusta University, Augusta, GA, USA; ^2^Janssen Research & Development, Spring House, PA, USA

**Keywords:** immunotherapy, Wnt, β-catenin, dendritic cells, Immune-tolerance, Immuno-oncology, autoimmune diseases, antitumor immunity

## Abstract

The Wnt/β-catenin pathway is an evolutionarily conserved signaling pathway critical for several biological processes. An aberrant Wnt/β-catenin signaling is linked to several human diseases. Emerging studies have highlighted the regulatory role of the Wnt/β-catenin signaling pathway in normal physiological processes of parenchymal and hematopoietic cells. Recent studies have shown that the activation of Wnt/β-catenin pathway in dendritic cells (DCs) play a critical role in mucosal tolerance and suppression of chronic autoimmune pathologies. Alternatively, tumors activate Wnt/β-catenin pathway in DCs to induce immune tolerance and thereby evade antitumor immunity through suppression of effector T cell responses and promotion of regulatory T cell responses. Here, we review our work and current understanding of how Wnt/β-catenin signaling in DCs shapes the immune response in cancer and autoimmunity and discuss how Wnt/β-catenin pathway can be targeted for successful therapeutic interventions in various human diseases.

## Introduction

The immune system functions constantly to defend from pathogens while preserving tolerance to self-antigens and commensal microbes (1, 2). Activation of the immune system in response to pathogens or sterile inflammation involves a cascade of innate and adaptive immune responses that are precisely controlled through a complex network of regulatory mechanisms (3, 4). Aberrations in immune-regulatory pathways result in different conditions including cancer and autoimmune diseases. The immune system is equipped with diverse cell types and secreted factors to achieve constant surveillance against pathogens while maintaining tolerance to self. Proinflammatory leukocyte subsets, such as monocytes, NK cells, and cytotoxic T cells, mainly provide protection from pathogens and cancer, whereas various subsets of regulatory T cells check untoward inflammatory mechanisms. Unlike other leukocytes, dendritic cells (DCs) possess the ability to elicit both pro- and anti-inflammatory functions made possible by the presence of various regulatory mechanisms, including crosstalk between Toll-like receptor (TLR)- and Wnt/β-catenin signaling pathways.

Dendritic cells are crucial in the induction of immunity and tolerance. Every tissue in humans is armed with DCs to sense and induce inflammatory immune responses against pathogens or to preserve a tolerogenic environment under steady state conditions. DCs equipped with pattern-recognition receptors (PRRs) play a central role in detection of pathogen-associated molecular patterns (PAMPs) and induction of robust host immunity through a controlled activation of different arms of the innate and adaptive immune responses (5, 6). DCs as professional antigen-presenting cells (APCs) recognize PAMPs through membrane-bound TLRs and C-type lectin receptors (CLRs) or cytoplasmic NOD-like receptors (NLRs) and RIG-1-like receptors (RLRs) (2, 3, 4).

Recognition of pathogens by DCs leads to phagocytosis of microbes, cytokine production, and expression of antigen presentation and costimulatory molecules, ultimately causing selective polarization of adaptive immune responses based on the type of pathogen. Accordingly, intracellular infections promote IFN-γ and IL-12 production by DCs that activates IFN-γ-producing CD4^+^ T helper 1 (Th1) and CD8^+^ cytotoxic T cell responses, whereas extracellular pathogens activate DCs to induce IL-4-producing CD4^+^ T helper 2 (Th2) cell responses (4, 7). Alternatively, DCs can induce IL-17A^+^CD4^+^ T helper 17 (Th17) cell responses against fungi and extracellular bacteria through increased production of IL-6, TGF-β, and IL-23 (3, 4, 8).

In addition to induction of immunity against pathogens, DCs contain effector immune responses and maintain peripheral tolerance to self-antigens, commensal microorganisms, and dietary components (2, 6). DCs generate tolerance and control chronic inflammatory responses by various mechanisms, including induction of anergic or regulatory T cell responses, and regulating effector Th1, Th2, and Th17 cell responses through differential expression of costimulatory molecules and secretion of regulatory cytokines (2, 6). Although significant progress has been achieved in our understanding on DCs’ diverse mechanisms in eliciting effector T cell responses in immunity, the signaling networks that program DCs into a regulatory state are poorly defined. In this regard, recent studies have discovered the critical functions of the Wnt/β-catenin signaling pathway of DCs in regulation of immune responses in various pathological conditions. Here, we review our current understanding and significance of Wnt/β-catenin signaling in DCs in the induction of immune tolerance in physiological conditions, autoimmuninty, and oncogenesis.

## Wnt/β-Catenin Signaling

**Figure 1:**
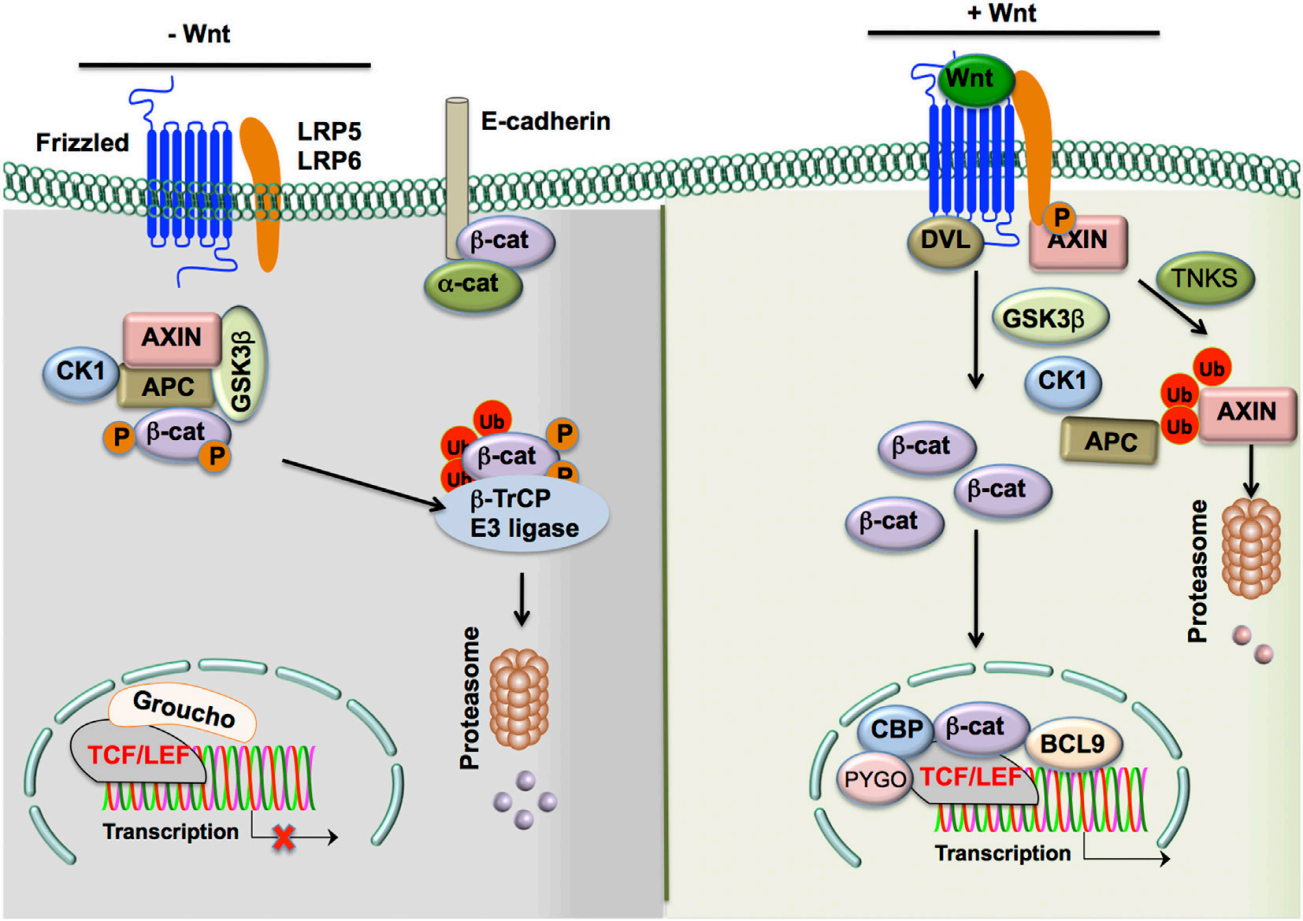
**The Wnt/β-catenin signaling pathway**. In the absence of Wnt ligands, β-catenin levels in the cytoplasm are largely reduced by the following means: (1) a destruction complex consisting of the scaffolding proteins APC and AXIN, as well as Ser/Thr kinases CK1α and GSK3β, interacts with β-catenin in the cytosol to induce phospho-inactivation of the protein by the two Ser/Thr kinases. (2 and 3) Phospho-β-catenin is promoted to ubiquitination and subsequent proteasomal degradation by β-Trcp and WTX proteins. In contrast, in the presence of Wnt ligands, β-catenin levels in the cytoplasm are upregulated and actively participate in cell signaling through the following steps: (1) Wnts bind to cognate Fzd receptors and interact with adjacent LRP5/6 co-receptors, (2) the resulting complex recruits DVL to the cytoplasmic tail of Fzd receptors, and (3) DVL recruits the destruction complex to the Fzd/LRP5/6 complex and associated Ser/Thr kinases such as GSK3β phosphorylate the co-receptor instead of β-catenin due to the altered localization of the complex. Moreover, cytoplasmic TNKS ubiquitinates Axin, targeting it for proteasomal degradation and causing disruption of the β-catenin destruction complex. (4) Cytosolic β-catenin levels are free to accumulate and subsequently translocate to the nucleus. (5) This translocation results in the displacement of the co-repressor Groucho on the TCF/LEF transcription factor by β-catenin and the recruitment of co-activators such as BCL9, CBP, and PYGO to result in the transcription of target genes. Alternate means of activation of β-catenin signaling include the disruption of epithelial cadherin (E-cadherin)–E-cadherin interactions, which frees the association of β-catenin to the cytoplasmic domain of E-cadherin and allows for its accumulation in the cytoplasm. In addition, TLRs and other such transmembrane receptors may result in PI3K/Akt signaling. This leads to an alternative phospho-activation of β-catenin through phosphorylation at Ser552 and phospho-inactivation of GSK3β at Ser9 to prevent the degradation of β-catenin by the destruction complex and allow it to accumulate in the cytoplasm and participate in cell signaling. Abbreviations: APC, adenomatous polyposis coli; CK1α, casein kinase 1α; GSK3β, glycogen synthase kinase 3β; WTX, Wilms tumor suppressor protein complex; β-Trcp, β-transducin repeat-containing protein; TNKS, tankyrase-1; Fzd, Frizzled; LRP5/6, low-density lipoprotein receptor-related protein 5/6; DVL, disheveled; TCF/LEF, T-cell factor/lymphoid enhancer factor; BCL9, B-cell CLL/lymphoma 9 protein; CBP, CREB-binding protein; PYGO, Pygopus; TLR, Toll-like receptor; PI3K, phosphoinositide-3-kinase.

Wnt/β-catenin signaling plays a critical role in cell differentiation, growth, proliferation, survival, and immune cell function (9, 10). Wnts are secreted lipid-modified glycoproteins that bind to seven-pass transmembrane Frizzled (Fzd) family receptors and activate multiple signaling pathways. In humans, there are 19 Wnt proteins and 10 cognate Fzd receptors (10, 11). The binding of Wnts to Fzd receptors activates canonical and/or non-canonical signaling pathways (Figure 1) (9, 10, 12). Low-density lipoprotein receptor-related protein 5 (LRP5) and LRP6 co-receptors are essential for optimal activation of canonical Wnt/β-catenin signaling (10, 13, 14). Wnt interaction with Fzd receptors results in cytoplasmic accumulation of β-catenin and its translocation into the nucleus, where it interacts with T-cell factor/lymphoid enhancer factor (TCF/LEF) and regulates the transcription of several target genes (9, 12). Wnt ligands also can stimulate other non-canonical signaling pathways independent of β-catenin activation (11).

In the absence of Wnt and Fzd interaction, β-catenin actively synthesized in the cytoplasm is removed through a destruction complex (9, 10, 14). This destruction complex consists of scaffolding proteins, such as axin and adenomatous polyposis coli (APC), and Ser/Thr kinases such as casein kinase 1α (CK1α) and glycogen synthase kinase 3β (GSK3β) (9, 10, 13). GSK-3β phosphorylates β-catenin, leading to ubiquitination and proteasomal degradation mediated by β-transducin repeat-containing (β-TrCP) protein and Wilms tumor suppressor protein complex (Wtx) (9–11). Wnt binding of the Fzd receptor leads to recruitment of co-receptors LRP5/6 and is followed by recruitment of disheveled (DVL) to the cytoplasmic tail of LRP5/6 (9, 10, 11, 13). These signaling events inactivate the destruction complex by recruiting it to the plasma membrane. Moreover, GSK3β in the destruction complex phosphorylates LRP5/6 instead of β-catenin, leading to increased accumulation of β-catenin in the cytoplasm (9, 10, 11, 13).

Thus, Wnts activate β-catenin signaling through inhibition of β-catenin destruction, resulting in its cytoplasmic accumulation and translocation to the nucleus. In the nucleus, β-catenin displaces the co-repressor molecule Groucho from the TCF/LEF complex and activates transcription of target genes (9, 10, 11, 13).

## Wnt-Independent β-Catenin Signaling in DCs

### E-Cadherin in β-Catenin Activation

**Figure 2:**
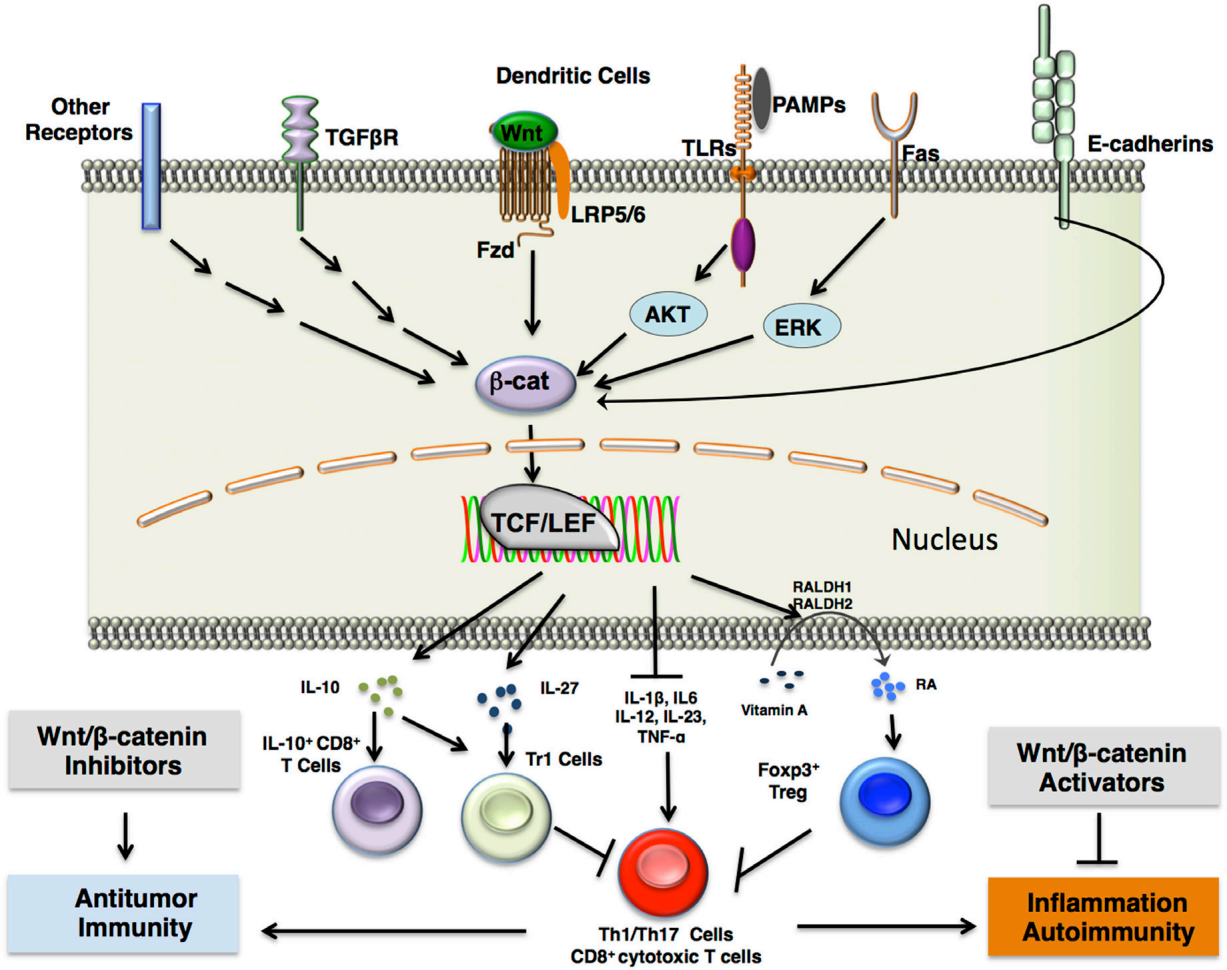
**β-catenin signaling in DCs induces immune tolerance**. β-catenin upregulation is induced in dendritic cells *via* a number of different receptor interactions, including canonical Wnt/Fzd signaling, stimulation of TLRs such as TLR2 with antigen and subsequent ERK signaling, Fas receptor activation, release of β-catenin from E-cadherins upon disruption of E-cadherin interactions, or activation of other pattern-recognition receptors such as Fcγ receptors or dectin-1. This results in β-catenin-mediated association of the TCF/LEF transcription factor with various co-activators that lead to transcription of target genes. This further results in the expression of anti-inflammatory cytokines, such as IL-10 and IL-27, which induce type 1 regulatory T (Tr1) cells or CD8^+^IL-10^+^ T cells, and TGF-β, which induces FoxP3^+^ regulatory T (Treg) cells. In addition, β-catenin signaling in DCs upregulate vitamin A-metabolizing enzymes (RALDH 1/2), which convert vitamin A to retinoic acid (RA). RA induces Treg differentiation. Accumulation of Tregs in the tissue microenvironment may result in inhibition of proinflammatory Th1/Th17 cells or CD8^+^ cytotoxic T cells. Pharmacological inhibition of β-catenin signaling components may thus lead to downregulation of regulatory T cells to mount a more robust effector T cell response against chronic infections and tumors, which may utilize β-catenin signaling components to engage in immune surveillance evasion. Such inhibitors include TNKS inhibitors, XAV939 and JW55, and PORCN inhibitor, C59. In contrast, pharmacological activation of β-catenin signaling components may result in upregulation of regulatory T cells to dampen chronic inflammation and autoimmunity. Such activators include the Axin/β-catenin interaction disruptor SKL2001 and administration of canonical Wnts. Abbreviations: Fzd, Frizzled; TLR, Toll-like receptor; ERK, extracellular signal-related kinase; Muc, mucin; Gal3, galectin 3; TCF/LEF, T cell factor/lymphoid enhancer factor; IL-10, interleukin 10; IL-27, interleukin 27; FoxP3, forkhead box P3; TGFβ, transforming growth factor β; RALDH 1/2, retinaldehyde dehydrogenase 1/2; Th1/17 cells, T helper 1/17 cells; TNKS, tankyrase-1; PORCN, porcupine.

In addition to Wnt-mediated activation of β-catenin, there are numerous pathways that function independent of Wnt ligands involved in activation of β-catenin in DCs (Figure 2). For example, disruption of E-cadherin–homophilic interactions in DCs activates β-catenin, which in turn programs DCs to a regulatory state (15). Importantly, these regulatory DCs produce high levels of IL-10 in response to LPS and protect mice from experimental autoimmune encephalomyelitis (EAE) (15). The cytoplasmic domain of E-cadherin is known to interact with and sequester β-catenin (16). This would therefore limit β-catenin signaling in a manner independent of Wnt ligands or axin destruction complex expression. E-cadherin-mediated disruption of β-catenin signaling is further supported by observations of phosphorylation and degradation of β-catenin by destruction complexes localized primarily at adherens junctions (17). This suggests a significant link between E-cadherin interactions and canonical Wnt signaling.

Additional binding factors also may mediate this function of E-cadherins at adherens junctions. Caveolin-1, for example, may be recruited by E-cadherin in order to support inhibition of β-catenin signaling (18). In a recent study, casein kinase 1 (CK1) was shown to phosphorylate another E-cadherin-associated protein known as p120-catenin, which in response to Wnt3a binding stabilizes E-cadherin interactions (19). This subsequently aids in recruitment of CK1 to the LRP5/6 co-receptor for canonical Wnt signaling pathway activation (19). In addition, CK1 phosphorylation of E-cadherin weakens its affinity for β-catenin, thus increasing free β-catenin levels in the cytosol for signaling. Elimination of p120-catenin inhibited Wnt-mediated β-catenin signaling. Unligated E-cadherin may therefore support β-catenin signaling by helping canonical Wnt signaling in cells. This indicates that the extent of cell–cell interactions mediated by E-cadherins does play a significant role in immunity and tolerance.

### TLRs in β-Catenin Activation

Activation of TLRs is known to modulate β-catenin signaling in different immune and non-immune cells (20). In this regard, a recent study has shown that TLR2 signaling through the PI3K/Akt pathway activates β-catenin in DCs and induces the expression of vitamin A-metabolizing enzymes and IL-10 (Figure 2) (21). Interestingly, activation of TLR2 in DCs promotes T regulatory cell responses and protects mice from Th1/Th17-mediated autoimmune neuroinflammation (21, 22). Stimulation of TLR2 present on DCs increased IL-10 production through increased expression and phosphorylation of extracellular signal-regulated kinase (ERK) and mitogen-associated protein kinase (MAPK) (23, 24). Phosphorylation of β-catenin at Ser552 by Akt activates its nuclear transcription activity, whereas Akt phosphorylation of GSK3β at Ser9 prevents it from marking β-catenin for degradation (25, 26, 27). Inhibition of the Akt or Erk pathway individually in DCs showed that β-catenin signaling *via* TLR2 stimulation is primarily dependent on Akt but not Erk (21). This shows that β-catenin signaling works synergistically with other pathways, such as the Erk pathway, to induce anti-inflammatory cytokines and proliferation of T regulatory cells. Like TLR2, other TLRs such as TLR3, TLR5, and TLR9 are known to activate or regulate β-catenin signaling *via* a mechanism dependent on PI3K/AKT and Erk pathways (20, 21, 28).

In addition to E-cadherin and TLR stimulation, other pathways such as FAS (29), TGF-β (30), and PLC-γ2 (31) activate or regulate β-catenin signaling in DCs. Further studies are warranted to elucidate how these pathways may work in tandem to effectively establish a tolerogenic phenotype in DCs.

## Wnt/β-Catenin Signaling of DCs in the Regulation of Tolerance and Inflammation

Although activation of effective innate and adaptive immune responses is critical for protective immunity, uncontrolled activation of these immune responses results in chronic autoimmune and inflammatory conditions. Moreover, induction of adaptive immunity against commensal microorganisms at mucosal sites, self-antigens, and food constituents is detrimental to human health (1, 2, 6). In this context, DCs play an important role in inducing peripheral immune tolerance at mucosal surfaces through several regulatory mechanisms (1, 2, 4, 6). Furthermore, DCs contribute to the resolution of inflammation through inhibition of ongoing effector T cell responses and induction of regulatory T cell responses. Although past studies have characterized the role of Wnt/β-catenin signaling in hematopoiesis, cell differentiation, proliferation, and homeostasis, the role of this pathway in DCs in regulating immune responses is poorly understood. Recently, several studies from others and our lab have begun to explore the function of this regulatory pathway in inducing immune tolerance and its significance during cancer, autoimmunity, and infectious diseases (Figure 2). In the next few sections, we will review recent developments in our understanding of the role of Wnt/β-catenin signaling in regulating immune responses in pathological conditions.

## Wnt/β-Catenin Signaling in DCs Regulates Mucosal Tolerance

Mucosal tolerance to commensals and food antigens is required for optimum health. Mucosal DCs play an important role in inducing peripheral tolerance. Gut DCs are in constant contact with microbial and food antigens and exhibit impaired immune-stimulatory capacity (2, 4, 6). Wnt/β-catenin signaling is essential for intestinal homeostasis including epithelial cell proliferation and maintenance of stem cells (32–36). Studies have shown that aberrant activation of the Wnt/β-catenin pathway in intestinal epithelial cells results in uncontrolled proliferation, polyps formation, and the development of colorectal cancer (33, 35, 37, 38, 39). Under homeostasis, the intestine expresses several components of the Wnt/β-catenin signaling pathway, including different Wnt ligands, Fzd receptors, and LRP5/6 co-receptors (40).

Although several studies have shown the importance of Wnt/β-catenin signaling in the maintenance of intestinal epithelial cell homeostasis, the role of this pathway in inducing immune tolerance is poorly defined. In this regard, a recent study showed that β-catenin is constitutively active in intestinal DCs and macrophages compared to splenic DCs (41). This study demonstrated that expression of β-catenin in intestinal DCs is critical for the induction of regulatory T cell responses and suppression of Th1 and Th17 cell responses. Thus, DC-specific deletion of β-catenin results in increased Th1 and Th17 cells over T regulatory cells in the intestine but not in the spleen (41). Interestingly, the absence of β-catenin in DCs shifts the balance from anti-inflammatory cytokine production to increased production of proinflammatory cytokines in the intestine, suggesting a tolerogenic role of β-catenin signaling in gut DCs (41). Moreover, a recent study showed that MUC2, a gel-forming mucin secreted by intestinal goblet cells, mediates mucosal tolerance in the intestine through activation of β-catenin signaling in DCs (42). This study showed that MUC2 is readily taken in by small intestinal DCs through interaction with Galectin-3, dectin-1, and FcγRIIB. This activates β-catenin in DCs to induce mucosal tolerance against intestinal commensal microorganisms. Thus, MUC2-mediated activation of β-catenin in small intestinal DCs suppressed inflammatory immune responses through suppression of the NF-κB pathway (42). These studies indicate the vital role of β-catenin signaling not only in epithelial cell homeostasis but also in the maintenance of gut tolerance through regulatory effects of DCs. Further studies on the role of commensal-derived factors, Wnt ligands, mucosal proteins, and various dietary ligands on activation of this pathway will identify novel regulatory mechanisms by which a normal intestine maintains tolerance. These studies will also help in designing better DC-based immunotherapy treatment approaches for enteric pathogens as well as chronic inflammatory conditions of the gut.

## Wnt/β-Catenin Signaling in DCs: A Potential Therapeutic Target for Chronic Inflammation

Although mucosal tolerance is actively enforced all the time to achieve optimum health, dysregulation of the host immune system and commensal microflora results in the development of inflammatory bowel diseases (IBDs) such as ulcerative colitis and Crohn’s disease (43). In IBD, intestinal DCs and macrophages lose their tolerogenic properties, resulting in uncontrolled intestinal inflammation (43). Accordingly, our recent study showed that mice lacking β-catenin in DCs are more susceptible to dextran sodium sulfate (DSS)-induced colitis, a mouse model for IBD, compared to β-catenin-sufficient mice, indicating the critical role of this pathway in regulation of inflammatory diseases (41). DC-specific deletion of β-catenin in mice led to increased body weight loss in response to DSS treatment with severe colonic epithelial layer destruction and increased Th1 and Th17 cell responses (41). Although this study identified the immune-regulatory role of β-catenin in DCs in inducing mucosal tolerance and protection against IBD, further studies are warranted to determine upstream and downstream signaling components for β-catenin activation in intestinal DCs.

Multiple sclerosis (MS) is a chronic autoimmune neuroinflammatory disease that leads to multifocal demyelination in the white matter of the human CNS with debilitating motor and sensory dysfunction (44, 45). Previous studies using EAE, a mouse model for MS, have shown that DCs play a critical role in initiation and development of CNS pathology through activation of effector T cells in the draining lymph nodes (DLN) and CNS (44, 45). These studies have shown that DCs contribute to CNS pathology through differentiation and activation of naive CD4^+^ T cells to myelin-specific effector Th1 and Th17 cells (44, 46). Furthermore, studies have also indicated the critical role of CD8^+^ T cells in causing EAE and MS pathogenesis (47, 48). Although the role of DCs in initiating effector T cell responses during EAE and MS is well characterized, emerging evidence indicates that DCs are also critical in resolving inflammation and in limiting immune-mediated pathology in EAE. DCs play a regulatory role through production of immune-regulatory factors, T regulatory cell activation, and fine-tuning of effector Th1 and Th17 cell responses (2, 4, 6). However, the molecular signaling networks and counter-regulatory mechanisms that induce these regulatory responses are poorly defined. Particularly, the immune modulatory factors in the local DLN and tissue microenvironment that are critical in programing DCs to induce tolerance during EAE and MS remain to be understood.

Recent studies from our laboratory have identified the critical function of Wnt/β-catenin signaling and the role of TLR2-mediated activation of β-catenin in regulating ongoing effector immune responses and CNS pathology (21, 49). Our data suggest that excessive neuroinflammation is controlled through increased Wnt ligand expression as a feedback mechanism to counter ongoing inflammatory responses. Thus, Wnt3a and Wnt5a expression is significantly upregulated in the DLN during the EAE induction phase and in the CNS during the effector phase. Interestingly, previous studies have shown that the dysregulated Wnt signaling leads to neurodegenerative and inflammatory disorders (50). Furthermore, it has been reported that sustained activation of the Wnt pathway restrains inflammation, stimulates neuroprotection, and promotes neurogeneration (49, 50). However, the role of Wnt/β-catenin in specific cell types has not been identified during ongoing EAE pathology. Results from our study show that DC-specific deletion of LRP5/6 or β-catenin leads to early onset as well as increased EAE pathology, indicating the critical role of canonical Wnt-mediated β-catenin activation in DCs in limiting CNS pathology (21, 49). Mechanistically, the absence of Wnt/β-catenin signaling in DCs lead to an increased proinflammatory cytokine production in the DLN and CNS, as well as increased effector Th1, Th17, and CD8^+^ T cell responses and diminished production of anti-inflammatory cytokines such as IL-10, TGF-β, and IL-27 (49). Furthermore, transgenic mice with a constitutively active form of β-catenin in DCs showed diminished neuroinflammation with reduced effector and increased regulatory T cell responses confirming the regulatory role of β-catenin signaling in DCs. Moreover, prophylactic and therapeutic treatment with β-catenin agonists in EAE-induced mice showed diminished EAE pathology, suggesting that immunotherapies targeting Wnt/β-catenin signaling specifically in DCs could represent a promising therapeutic approach for clinical management of MS.

Interestingly, Wnt5a, which signals independently of the β-catenin pathway, also reprograms DCs to limit the expression of inflammatory cytokines (51, 52). It is possible that both the canonical and non-canonical pathways might act in concert to regulate the level of inflammation. In addition to direct Wnt-mediated activation of β-catenin and other regulatory pathways, TLR2-mediated signals also induce active β-catenin in DCs and regulate EAE pathology (21). This study showed that TLR-2-mediated activation of β-catenin in DCs promotes IL-10 and retinoic acid (RA) production, which in turn promotes regulatory T cell responses and suppresses effector T cell responses. Furthermore, E-cadherin-mediated signaling can also activate β-catenin in DCs, suppressing chronic inflammation and EAE disease severity (15, 21). In addition to DCs, macrophages and microglial cells also play a critical role in initiation and resolution of EAE pathogenesis (53, 54). Although these observations indicated the role for Wnts and TLRs in modulating DC activity during EAE, it is quite possible that increased Wnt ligand expression could regulate functions of macrophage and microglial cells and limit EAE pathology. Further studies on cellular source and targets of Wnt ligands during different stages of EAE are necessary to delineate the regulatory role of the Wnt/β-catenin pathway during autoimmune neuroinflammation.

Several recent studies have characterized the critical role of Wnt/β-catenin signaling in the pathogenesis of chronic inflammatory diseases such as lupus, psoriasis, and rheumatoid arthritis. For example, using murine models for lupus, it has been shown that Dickkopf-1 (DKK1), a negative regulator of the Wnt/β-catenin pathway, is highly upregulated in the serum during lupus progression (55). Similarly, serum samples from lupus patients showed increased levels of DKK1, suggesting altered canonical Wnt signaling in the development of lupus (56). DKK1 overexpression has also been observed in humans in other autoimmune disorders. For example, skin and peripheral blood mononuclear cells expressed increased levels of the Wnt signaling inhibitor in psoriasis (57). However, it is not known whether increases in the levels of negative regulators of the Wnt pathway alter functions of DCs or macrophages.

In contrast to negative regulators of the canonical Wnt-pathway, aberrant expression of Wnts such as Wnt5a and Wnt7B that activate the non-canonical pathway was observed in the joints of rheumatoid arthritis patients (58). Conversely, effects of these Wnts on immune cell function are not known. Recent studies also imply downregulation of canonical Wnt signaling receptors in rheumatoid arthritis, as indicated by silencing of Fzd8 by miRNA-375 in arthritis synovial fibroblasts (59). Activation of Wnt signaling in rheumatoid arthritis has been known to induce proliferation of fibroblast-like synoviocytes and increases in synovial inflammation (58). Also, non-canonical pathways may antagonize the canonical β-catenin signaling pathway by downregulating β-catenin protein expression (60, 61). Thus, it appears that upregulation of non-canonical Wnt signaling that leads to downregulation of canonical Wnt signaling may be occurring in rheumatoid arthritis to induce high levels of synovial inflammation. Wnt signaling has been known to play a role in cell renewal and keratinocyte differentiation, and the Wnt/Ca^2+^ non-canonical pathway has been correlated with the pathophysiology of psoriasis (62). The overexpression of Wnt5A and Fzd5 in psoriasis may denote the targeting of non-canonical Wnt pathway elements as an efficacious tactic in the remediation of psoriatic symptoms. Thus, activating the canonical Wnt/β-catenin pathway and suppressing non-canonical Wnt signaling represent a potential therapeutic approach toward suppressing inflammation and limiting immune-mediated pathology.

## Wnt/β-Catenin Signaling in DCs Modulates Antitumor Immunity

Although activation of β-catenin signaling in DCs is critical for mucosal tolerance and resolving chronic inflammatory conditions, tumor cells, and some pathogenic microbes exploit this pathway to effectively suppress DC-mediated host immune responses (20, 21, 63). Tumors use different mechanisms to evade immunity, including suppressing DC-induced antitumor immune responses in the tumor microenvironment (TME) (64, 65). Numerous studies have shown increased Wnt expression in tumors and linked dysregulation of the Wnt pathway with tumor progression (66). However, most studies focused on how the Wnt signaling cascade regulates tumor development, progression, and metastasis (67–70). The role of Wnt/β-catenin signaling in immune cells in the TME and its effect on host antitumor immunity and tumor-induced immune tolerance remain unknown. Recent studies from several labs have shown that increased levels of Wnts in the TME can initiate paracrine signaling and modulate host antitumor immunity (63, 71–75).

Using different murine tumor models, we have shown that increased Wnt ligands in tumors program DCs to produce RA and IL-10, which promotes immune suppression by inducing regulatory T cell responses (63). Mechanistically, this is also due to activation of β-catenin signaling in TME DCs and their ability to up regulate immune-regulatory factors such as IDO, IL-10, and Raldh (63, 71, 73, 75). Furthermore, TME DCs express high levels of IL-10 and TGF-β that promotes immune tolerance through induction of IL-10-producing CD4^+^ and CD8^+^ T cells (63). Interestingly, IL-10 production by TME DCs is dependent on the β-catenin/TCF4 signaling axis (63, 71, 75, 76) and mTOR/β-catenin signaling pathway (71). These studies further suggest that Wnt-mediated activation of β-catenin in TME DCs is critical for inducing tumor tolerance. Accordingly, conditional deletion of β-catenin or LRP5/6 in DCs markedly reduces regulatory T cell responses with increased effector CD8 T cell responses, leading to suppression of tumor growth (63, 75). Furthermore, blocking Wnt interaction with LRP5/6 and Fzd using small molecule inhibitors in tumor-bearing mice showed increased capture of tumor-associated antigen by DCs. This led to increased cross-priming of CD8 T cells and resulted in reduced tumor burden. Interestingly, recent study showed that melanoma-intrinsic β-catenin signaling suppresses antitumor immunity by regulating DC migration (74).

In addition, the TME also programs DC function by regulating its activation and maturation through diverse mechanisms, including expression of costimulatory (CD80, CD86, and CD40) and co-inhibitory (PD-L1 and PD-L2) molecules (64). Our data show that deletion of β-catenin in DCs results in increased activation of DCs in the TME with increased surface expression of costimulatory molecules and decreased expression of co-inhibitory molecules (63). In addition to inducing regulatory T cells, β-catenin activation in DCs can also affect cross-priming of CD8^+^ T cell responses against tumors (71, 75). Though β-catenin is known to suppress DC activation and cross-priming (77), it remains to be determined whether β-catenin directly regulates DC activation or does so indirectly *via* RA and IL-10 (63).

Since Wnt-mediated activation of β-catenin in the TME promotes tumor progression through multiple mechanisms, pharmacological suppression of the β-catenin pathway represents a promising target for effective antitumor immunotherapy. Accordingly, treatment of mice with inhibitors that blocks the β-catenin/TCF pathway results in delayed tumor growth through suppression of regulatory T cell responses and increased antitumor CD4 and CD8 T cell responses (63, 71–73, 75). Collectively, these observations suggest that Wnt family of ligands in the TME can initiate tumor-intrinsic β-catenin signaling and DC-intrinsic β-catenin signaling, and both contributing to immune suppression through different effector mechanisms. Blocking the Wnt/β-catenin pathway could represent a promising therapeutic approach toward breaking tumor-mediated immune suppression and augmenting antitumor immunity.

## Regulation of Wnt/β-Catenin Signaling in DCs: Novel Targets for Immunotherapies

**Figure 3:**
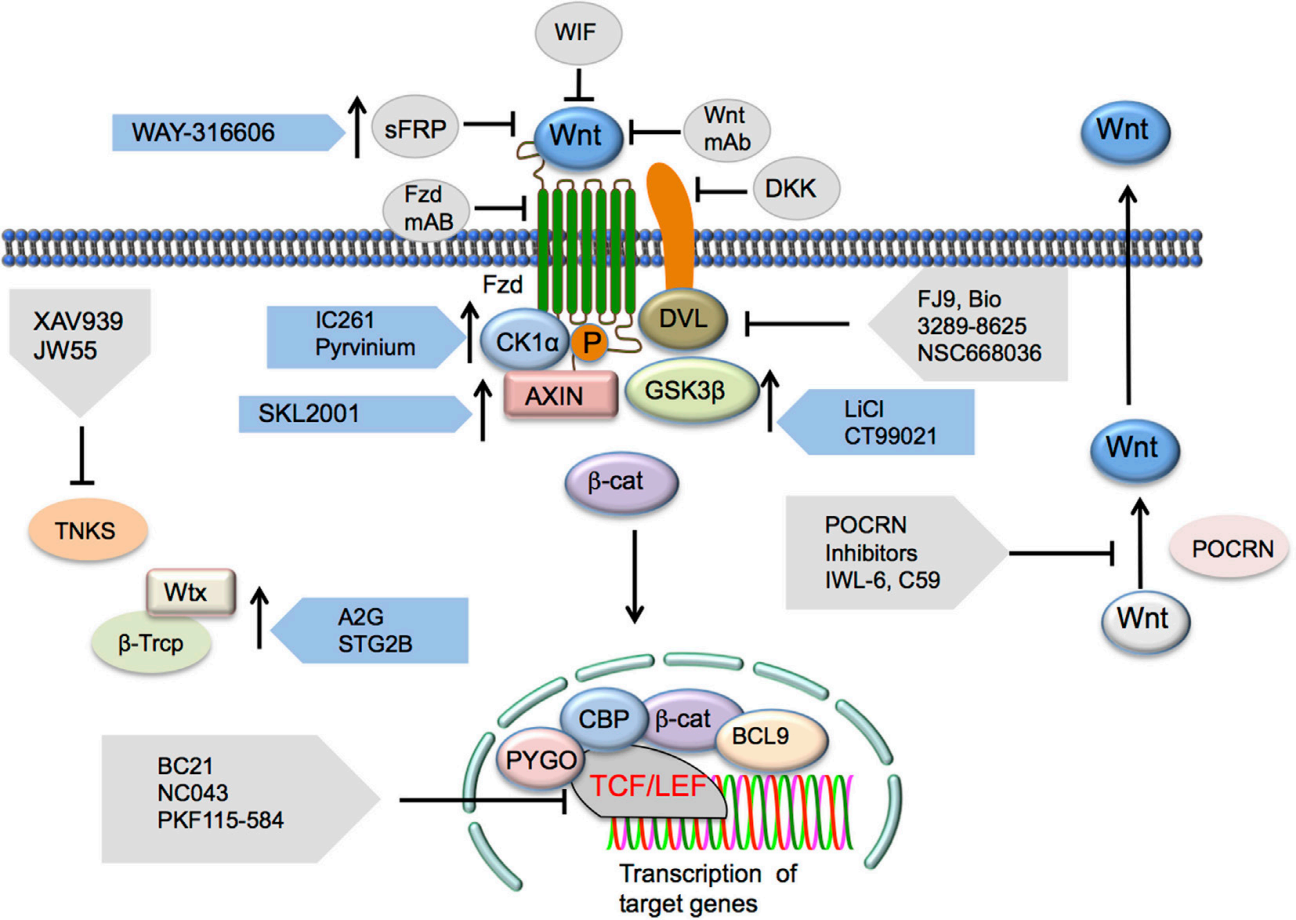
**Potential Wnt/β-catenin signaling therapeutic targets**. Inhibition of the canonical Wnt/β-catenin pathway may occur *via* targeting a number of the pathway’s elements. Wnt ligand antagonists include SFRP, WIF, or mAbs generated against the ligands. Frizzled receptor interaction inhibition may occur through the administration of Fzd mAbs as well. DKK1 has been shown to elicit inhibition of LRP co-receptor function. Disheveled (DVL) interactions with Wnt signaling receptors may also be inhibited *via* FJ9, Bio 3289-8625, or NSC668036. Downstream inhibition may occur through inhibition of β-catenin and TCF/LEF interactions by a number of different inhibitors. Other Wnt signaling-activating components that may be inhibited include TNKS, an inhibitor of axin stability, and PORCN, a stimulator of Wnt secretion. Inhibition of this pathway may also be the result of the presence of activators of elements that regulate the functions of this pathway that induce the upregulation or increase in function of these regulatory elements. Such activators include those targeting the Ser/Thr kinases CK1α (IC261 and pyrvinium) and GSK3β (lithium, CT99021, and BIO), the axin scaffolding protein (SKL2000), or β-Trcp (Δ2G, STG28). Abbreviations: SFRP, secreted frizzled-related protein-1; WIF, Wnt inhibitory protein; Fzd, Frizzled; mAbs, monoclonal antibodies; DKK1, Dickkopf-related protein 1; LRP, low-density lipoprotein receptor-related protein; TCF/LEF, T cell factor/lymphoid enhancer factor; TNKS, tankyrase 1; PORCN, porcupine; CK1α, casein kinase 1α; GSK3β, glycogen synthase kinase 3β; β-Trcp, β-transducin repeat-containing protein.

The Wnt/β-catenin pathway in DCs is a key player in the pathogenesis of a wide amalgam of inflammatory and infectious diseases (10). Wnt/β-catenin pathway thus serves as a potential therapeutic target where activation results in the suppression of inflammatory pathologies, and suppression is beneficial in augmenting antitumor immunity (Figure 2) (21, 49, 63, 71–73, 75). Small molecules that modulate the Wnt/β-catenin pathway have been extensively researched for use against various pathological conditions (70, 78) (Figure 3). In this regard, a recent study of ours has shown that pharmacological activation of canonical Wnt/β-catenin signaling is beneficial in reducing EAE and associated CNS pathology (49). Mechanistic investigations about various pathways related to Wnt signaling have provided additional therapeutic targets for activation or suppression of canonical Wnt signaling in diseases (Figure 3). In addition to direct inhibition or activation of the canonical Wnt pathway, suppression of non-canonical Notch signaling, which can antagonize canonical Wnt signaling, may be beneficial in regressing symptoms of autoimmune disease. Non-canonical Wnt ligands Wnt5a and Wnt7b were upregulated in the joints of rheumatoid arthritis patients, and the non-canonical Wnt/Ca^2+^ pathway has been associated with the pathophysiology of psoriasis (58, 62). Pharmacological inhibition of Wnt5A, for example, may remediate inflammation and other symptoms in arthritis or psoriasis patients.

Optimal functioning of Wnt/β-catenin pathway in different type of non-immune and immune cells including DCs is vital for a range of cellular functions and homeostasis. Consequently, targeting Wnt/β-catenin pathway specifically in DCs and particularly in those DCs, which play a central role in the induction of autoimmune pathology or tumor progression is critical for successful therapeutic intervention to minimize off-target side effects. Therefore, it is crucial to understand the biology of different subsets of DCs and how Wnt/β-catenin pathway in these DCs subsets contributes to normal health and immunopathology. Moreover, further studies on understanding the biology Wnt/β-catenin signaling in other immune cells such as B cells, CD8^+^ T cells and different subsets of CD4^+^ T cells are warranted for better therapeutic targeting of this pathway in autoimmunity and cancer. In this regard, recent advances in targeted delivery of small molecules and biologics represent a novel therapeutic approach to deliver Wnt/β-catenin signaling modulator specifically to DCs for clinical management of autoimmunity and cancer. Additionally, combining DCs-specific Wnt/β-catenin pathway inhibition with recent successful therapies targeting immune checkpoints represents a promising approach to promote antitumor immune responses.

## Summary

The normal immune system functions by instigating an effective immune response against infectious agents while promoting tolerance toward commensal microbiota and self-antigens. The Wnt/β-catenin pathway participates in this balance through diverse mechanisms including the generation of tolerogenic DCs to regulate inflammatory responses. Inadequacies in the signaling of the Wnt/β-catenin pathway may result in allergic and autoimmune diseases through chronic and uncontrolled activation of the immune system or cancer due to inadequate immune surveillance resulting from excessive immune tolerance. Thus, Wnt/β-catenin signaling serves as a molecular switch between opposing immune functions, and targeting different elements of this pathway may provide therapeutic benefits in the remediation of autoimmune diseases, cancer, and infectious diseases. While it is clear that Wnt-signaling programs DCs to induce robust regulatory T cell responses and cytokines to contain inflammation, several important questions remain unanswered. It is unknown, for example, how the canonical and non-canonical pathways act in tandem to regulate immunity and tolerance, and how much crosstalk is involved with other pathways involved in the regulation of immune responses. Dissecting many of these issues around Wnt/β-catenin signaling in immune cells potentially helps in developing novel therapeutic strategies against various diseases stemming from an imbalance in the immune system.

## Author Contributions

AS and RT have performed bibliographic researches and drafted the manuscript. DS has performed bibliographic researches. SM has performed bibliographic researches and drafted the manuscript.

## Conflict of Interest Statement

The authors declare that the research was conducted in the absence of any commercial or financial relationships that could be construed as a potential conflict of interest.
